# Time-Dependent Sensitivity Tunable pH Sensors Based on the Organic-Inorganic Hybrid Electric-Double-Layer Transistor

**DOI:** 10.3390/ijms231810842

**Published:** 2022-09-16

**Authors:** Ki-Woong Park, Won-Ju Cho

**Affiliations:** Department of Electronic Materials Engineering, Kwangwoon University, 447-1 Wolgye-dong, Nowon-gu, Seoul 139-701, Korea

**Keywords:** ion-sensitive field-effect transistor (ISFET), tunable sensitivity, organic–inorganic electric-double-layer transistor, chitosan electrolyte, extended gate, amorphous oxide semiconductor

## Abstract

In this study, we propose tunable pH sensors based on the electric-double-layer transistor (EDLT) with time-dependent sensitivity characteristics. The EDLT is able to modulate the drain current by using the mobile ions inside the electrolytic gate dielectric. This property allows the implementation of a device with sensitivity characteristics that are simply adjusted according to the measurement time. An extended gate-type, ion-sensitive, field-effect transistor consisting of a chitosan/Ta_2_O_5_ hybrid dielectric EDLT transducer, and an SnO_2_ sensing membrane, were fabricated to evaluate the sensing behavior at different buffer pH levels. As a result, we were able to achieve tunable sensitivity by only adjusting the measurement time by using a single EDLT and without additional gate electrodes. In addition, to demonstrate the unique sensing behavior of the time-dependent tunable pH sensors based on organic–inorganic hybrid EDLT, comparative sensors consisting of a normal FET with a SiO_2_ gate dielectric were prepared. It was found that the proposed pH sensors exhibit repeatable and stable sensing operations with drain current deviations <1%. Therefore, pH sensors using a chitosan electrolytic EDLT are suitable for biosensor platforms, possessing tunable sensitivity and high-reliability characteristics.

## 1. Introduction

pH biosensors are extensively used in various fields, such as disease diagnosis, soil or water quality measurements, the food industry, and chemical research (e.g., the optimal pH value of blood ranges from 7.35 to 7.45) [1,2,3]. In particular, the ion-sensitive field-effect transistor (ISFET)-based electrochemical pH sensor has been actively studied, owing to its compact size, complementary metal-oxide-semiconductor process compatibility, easy integration capability, low cost, and label-free detection of analytes [4,5]. The gate electrode of a typical ISFET is replaced with an ion-sensitive membrane and an electrolyte solution grounded by a reference electrode. In this structure, ions in the electrolyte bound to the ion-sensitive membrane change the surface potential of the membrane, which is transduced as a decipherable electrical signal through the FET [6,7]. In addition, many studies have reported on extended-gate ISFET (EG-ISFET)-based pH sensors in which the detector (sensing membrane) and the transducer (FET) are separated [8,9,10]. Conventional ISFETs have a problem in that the sensing membrane of the detector is damaged continuously when exposed to pH buffers. However, EG-ISFET provides a cost-effective sensor platform because it can easily replace damaged detectors and reuse transducers [11]. Meanwhile, electric-double-layer transistors (EDLTs) are drawing attention because they can accumulate high-charge-carrier densities by extremely strong EDL gating [12,13]. Mobile ions inside the gate insulating electrolyte readily migrate to the channel/electrolyte interface, owing to the gate bias to form an EDL, which functions as a nanogap capacitor, thus contributing to a high-gate capacitance and inducing a lower operating voltage [14,15]. Furthermore, the gradual migration and accumulation of mobile ions by the gate bias continuously alters the channel conductance of the FET [16]. Owing to these unique features, many studies have been conducted to apply EDLT to artificial synaptic devices or biochemical sensor platforms [17,18].

**Figure 1:**
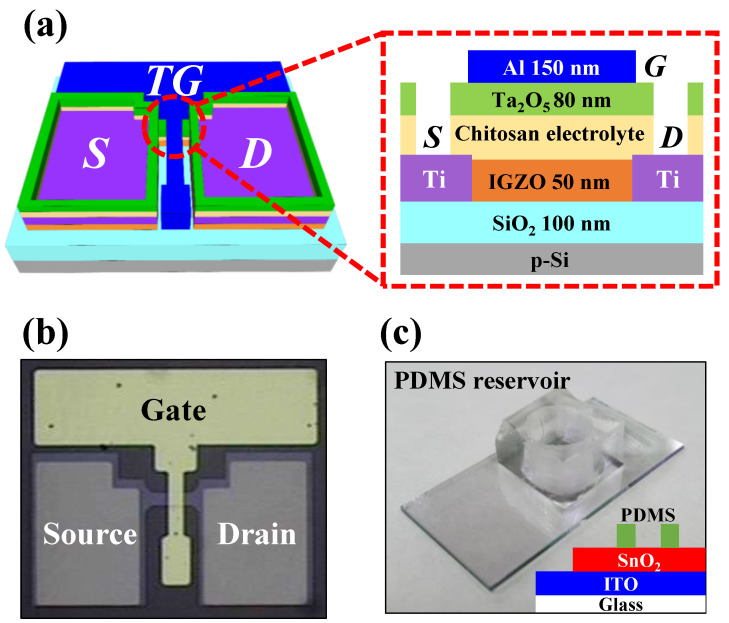
(**a**) Schematic in three-dimensions and in cross-section. (**b**) Optical microscopic image of the fabricated chitosan/Ta_2_O_5_ hybrid dielectric electric-double-layer transistor (EDLT). (**c**) SnO_2_ extended gate (EG) on ITO/glass substrate.

In this study, we propose a pH sensor platform and sensing mechanism with tunable sensitivity by using an EDLT as a transducer for EG-ISFETs. In practical applications, pH sensors with tunable sensitivity are advantageous for conducting measurements over a wide variety of pH ranges, either narrow or wide [19]. In general, ISFETs operating in the quasistatic mode have only a fixed sensitivity. In contrast, to achieve tunable sensitivity, many studies have been reported that have focused (among others) on additional configurations of the gate electrode or on different designs of the sensing area of the device [20,21,22]. However, these additional components adversely affect device integration and cost-effectiveness. The pH sensor platform that uses the EDLT proposed in this study is able to easily tune the sensitivity by only adjusting the measurement time. Given that the EG and the EDLT are electrically connected, the surface potential that depends on the pH level of the sensing membrane is delivered to the gate electrode of the EDLT. As a result, the migration of ions in the electrolyte depends on the pH level of the buffer, which causes a difference in the drain current of the EDLT in response to the pH level. By utilizing these distinctive characteristics, it is possible to implement pH sensing and facile sensitivity tuning. We fabricated chitosan/Ta_2_O_5_ hybrid dielectric EDLTs and measured their basic electrical properties (Figure 1b). The drain current response of the pH sensor composed of the prepared EDLT and EG according to the pH level of the buffer solution was then measured. Finally, modulation of the drain current and tuning of the sensitivity by pulse duration were demonstrated as intended, achieving a tunable pH sensor. These distinctive operations of the pH sensor by using EDLT were verified by comparison with devices composed of a normal SiO_2_ dielectric FET with the same structure. In addition, the repeatability and stability of the sensing operation of the pH sensor using EDLT were evaluated.

## 2. Results and Discussion

**Figure 2:**
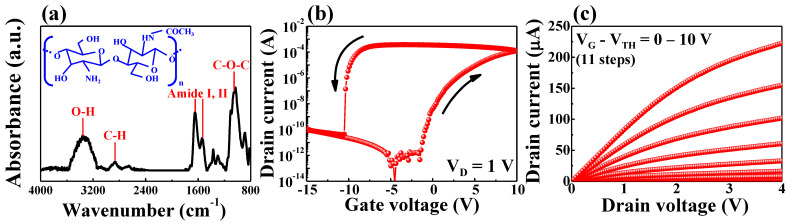
(**a**) Fourier transform infrared spectrum of the solid-state chitosan electrolyte; (**b**) transfer curves (I_D_–V_G_) in double-sweep mode; and (**c**) output curves (I_D_–V_D_) of the chitosan/Ta_2_O_5_ hybrid dielectric EDLT.

The Fourier transform infrared spectroscopy (FT-IR) outcome of the solid-state chitosan electrolyte, which plays a key role in EDLT, is shown in Figure 2a. The inset indicates the structure of the molecules constituting the chitosan electrolyte. Chitosan shows bands at approximately 3355 and 2860 cm^−1^ attributed to O–H and C–H stretching, respectively. Additionally, the bands at approximately 1645, 1529, and 1053 cm^−1^ were caused by the amide I, amide II, and C–O–C groups, respectively [23,24,25]. These groups facilitate the migration of hydrogen ions (protons) in the chitosan electrolyte [26]. The fundamental electrical characteristics of the chitosan/Ta_2_O_5_ hybrid dielectric EDLT are presented in Figure 2c. Figure 2b shows the transfer curve (I_D_–V_G_) measured by double sweeping of the gate voltage (V_G_) from -15 to 10 V at a constant drain voltage of V_D_ = 1 V. It can be observed that the transfer curve exhibits the counterclockwise hysteretic loop typically observed in electrolyte-based EDLTs, and has a hysteretic window of 10.1 V [27,28]. This hysteretic loop is induced by the slow polarization of mobile protons in the chitosan electrolyte [29]. When a positive voltage is applied to the gate electrode, protons in the chitosan electrolyte migrate to near the channel of the EDLT, thus inducing charge carriers and increasing the conductance of the channel. The migrated protons are fully depolarized, with a sufficient negative voltage in the backward sweep and return to their initial state. This hysteretic effect is a unique property rarely observed in typical FETs with SiO_2_ gate dielectrics, thus allowing EDLTs to mimic biological synaptic behavior. Figure 2c shows the output curve (I_D_–V_D_) measured by sweeping V_D_ from 0 to 4 V and V_G_–V_TH_ from 0 to 10 V. The I_D_ increased linearly in the low-V_D_ region and gradually saturated in the high-V_D_ region, thus indicating ohmic characteristics at the source/drain contact.

**Figure 3:**
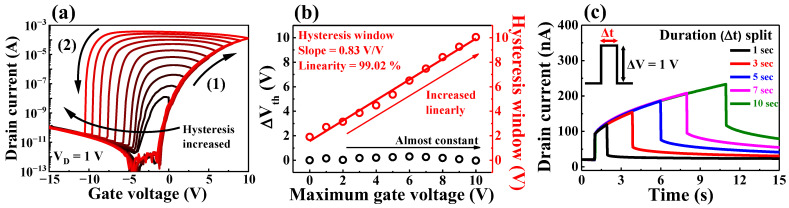
(**a**) Double-sweep transfer curves of the chitosan/Ta_2_O_5_ hybrid dielectric EDLT at increasing maximum V_G_; (**b**) threshold voltage and hysteresis window extracted from the transfer curves; (**c**) dynamic I_D_ responses to gate pulses of various durations.

For further investigation of the proton ionic polarization in chitosan electrolytes, transfer curves at various maximum V_G_ values were measured, and dynamic I_D_ responses to pulse durations were evaluated. As shown in Figure 3a, the double-sweep transfer curves were measured by increasing the maximum V_G_ from 0 to 10 V (in increments of 1 V). The threshold voltages (V_th_) and hysteretic windows extracted from the measured transfer curves are shown in Figure 3b. The hysteretic window linearly increased with a slope of 0.83 V/V, and a high linearity of 99.02% was achieved as the maximum V_G_ value increased. As the maximum V_G_ increases, more mobile ions migrate to the chitosan/channel interface, which requires an increased gate voltage to depolarize them again [30], thus resulting in a gradual increase in the hysteretic window. Meanwhile, V_th_ remained almost constant despite the increase in V_G_, owing to the full depolarization of protons by sufficient negative gate bias. Figure 3c shows the dynamic I_D_ response to a gate pulse (∆V = 1V) at various pulse durations from 1 to 10 s. Given that the ionic polarization of protons induces electrons into the channel, the I_D_ gradually increases while a positive gate pulse is applied. In addition, the peak value of I_D_ increased at longer pulse durations. In contrast, the characteristics of increasing hysteresis and I_D_ response were not observed in normal FETs of the SiO_2_ gate dielectric with only dipole polarization (Figure S2). These results indicate that the I_D_ of the chitosan/Ta_2_O_5_ hybrid dielectric EDLT changes depending on the amplitude and duration of the gate voltage.

**Figure 4:**
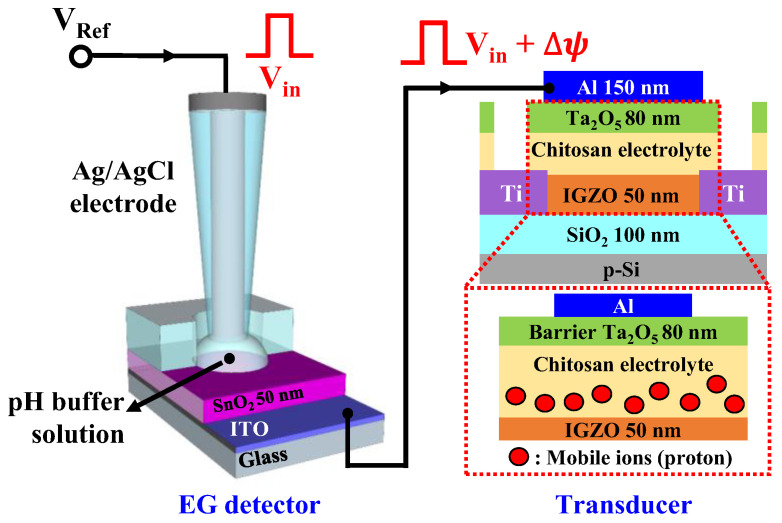
Schematic of a time-dependent pH sensor configured with a tunable chitosan/Ta_2_O_5_ hybrid dielectric EDLT and an EG.

Figure 4 shows a schematic of the proposed time-dependent sensitivity tunable pH sensing system consisting of an EG detector and a chitosan/Ta_2_O_5_ hybrid dielectric EDLT. In this sensing system, the electrical signal for pH sensing is sequentially transferred to the gate electrode of the EDLT through the Ag/AgCl reference electrode, the pH buffer solution, the SnO_2_ sensing membrane, and the ITO electrode of the EG. According to the site binding model, the surface potential (*ψ*) of the SnO_2_ sensing film can be determined by the chemical sensitivity of a given surface and the number of ions in the pH solution as shown in Equation (1) [31,32]:

(1) $$\psi =2.303\frac{kT}{q}\frac{\beta }{\beta +1}\left(pH_{pzc}-pH\right),$$

where *k* is the Boltzmann constant, *q* is the elementary charge, *T* is the absolute temperature, β is the chemical sensitivity of the sensing membrane, and *pH_pzc_* is the pH level at which the charge is zero. Additionally, this change in surface potential (∆*ψ*) arises depending on the pH level of the buffer solution. The most common sensing method, the quasistatic detection mode, detects the pH level through the transfer curve shift induced by ∆*ψ* of the FET [33,34].

Figure S3 shows the transfer curves and reference voltage shift (∆V_R_) for various pH buffer solutions. The transfer curve shifted in a positive direction as the pH decreased, regardless of the transducer. The pH sensitivities in quasistatic mode for the EDLT (with chitosan/Ta_2_O_5_ hybrid gate dielectric) and the normal FET (with SiO_2_ gate dielectric) were similar and equal to 50.17 and 51.72 mV/pH, respectively. Note that these sensitivities are fixed values for the same sensing system unless an additional parameter is provided, such as a second gate voltage. In contrast, the sensitivity can be easily changed in the dynamic sensing mode by using the EDLT and electrical pulses proposed in this study. Based on *ψ* for pH7, ∆*ψ* is a positive voltage in a cation-abundant acidic buffer solution, and a negative voltage in an anion-abundant basic buffer solution. A positive or negative surface potential is superimposed on the electrical sense pulse (V_in_), thus changing the amplitude of the effective pulse reaching the EDLT. Given that the I_D_ depends on the amplitude and duration of the gate pulse, as illustrated in Figure 3, the peak of the I_D_ is determined by the pH level and the duration of the sensing pulse. Owing to these unique features of the proposed sensing system, the chitosan/Ta_2_O_5_ hybrid dielectric EDLT-based pH sensor has tunable pH sensitivity by adjusting the duration of the gate pulse.

**Figure 5:**
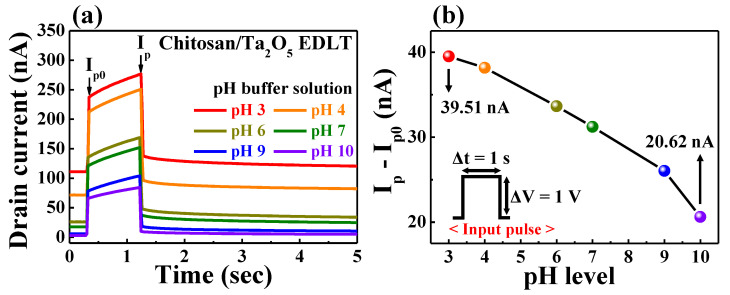
(**a**) Dynamic I_D_ response in various pH buffer solutions; (**b**) pH-dependent changes in I_D_ (I_p_−I_p0_) at a gate pulse (1s duration) of the chitosan/Ta_2_O_5_ hybrid dielectric EDLT.

To evaluate the pH-sensing behavior, we measured the dynamic I_D_ response of chitosan/Ta_2_O_5_ hybrid dielectric EDLT in various pH buffers. Figure 5a shows the I_D_ response of a chitosan/Ta_2_O_5_ hybrid dielectric EDLT upon application of a gate pulse with an amplitude (ΔV) of 1 V and a duration (Δt) of 1 s. Given that the IGZO channel of EDLT has n-type properties, the I_D_ response was higher in the cation-abundant acidic buffer (Δ*ψ* > 0) than in the anion-abundant basic buffer (∆*ψ* < 0) [35]. In addition, the I_D_ gradually increased owing to the continuous migration of protons during the pulse duration, and the difference in I_D_ depends on the pH level. Figure 5b shows the change in current between the start (I_p0_) and end (I_p_) of the I_D_ at a gate pulse duration of 1 s, whereby the I_p_ − I_p0_ value decreased from 39.51 nA (pH 3) to 20.62 nA (pH 10) as the pH increased. Although the amplitude and duration of the external pulses were the same, effective pulses of different amplitudes were entered into the transducer owing to the surface potential caused by the pH buffer solution. Meanwhile, the pH-level-dependent pulse changed the I_p_ − I_p0_ value (as documented by the I_D_ increasing rate with respect to the pulse duration). The longer the pulse duration is, the greater the difference is in the final I_p_ according to the pH level. This is an important property for adjusting the pH sensitivity in this study. Similarly, when a normal FET with a SiO_2_ gate dielectric was used as a transducer, the I_D_ response was higher in acidic solutions (Figure S4). However, except for a slight change in I_D_ induced owing to charge trapping, a normal FET without mobile protons has a constant I_p_ − I_p0_ value (~2.08 nA) close to zero, regardless of the pH value. To evaluate the pulse duration dependence in the pH sensing operation, external pulses (fixed amplitude) with different durations were applied to the Ag/AgCl reference electrode in three types of buffer solutions. Figure S5 shows the I_D_ responses in buffers with pH values of 3, 7, and 10, with a continuously increasing drain current as a function of pulse duration, thus reaching higher values in more acidic buffers.

**Figure 6:**
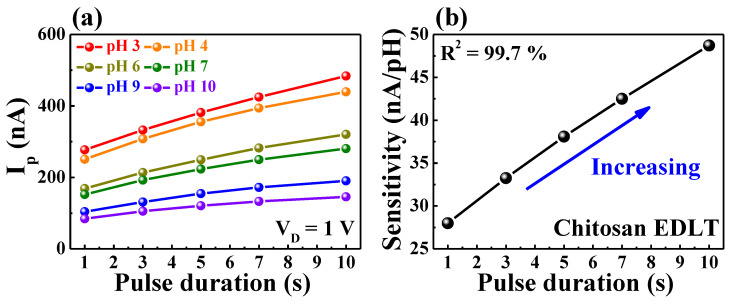
(**a**) Modulation of I_p_ for a gate pulse and (**b**) pH sensitivity as a function of the pulse duration of the chitosan/Ta_2_O_5_ hybrid dielectric EDLT in various pH buffers.

Figure 6a shows the modulation of I_p_ for gate pulses (∆V = 1 V) with different durations in various pH buffers. The I_p_ value was obtained from the end of I_D_ in terms of the gate pulse duration in each measurement; this value increased at lower pH levels and longer pulse durations. Figure 6b shows the pH sensitivity as the slope of I_p_ with respect to the pH level as a function of the gate pulse duration of the chitosan/Ta_2_O_5_ hybrid dielectric EDLT pH sensor. The pH sensitivities were 27.99, 32.25, 38.09, 42.49, and 48.71 nA/pH for pulse durations of 1, 3, 5, 7, and 10 s, respectively, amplified up to 174% without adding electrodes or changing the design of the device. In contrast, in the normal FET pH sensor with a SiO_2_ gate dielectric, a constant I_p_ determined by the pH level was observed, regardless of the pulse duration, as shown in Figure S6. Thus, a pH sensor configured by a normal FET has a fixed pH sensitivity of 43.39 nA/pH, a feature typically found in FET-type pH sensors. Accordingly, the time-dependent I_D_ modulation nature of EDLT enabled the implementation of a unique, sensitivity-tunable pH sensor.

**Figure 7:**
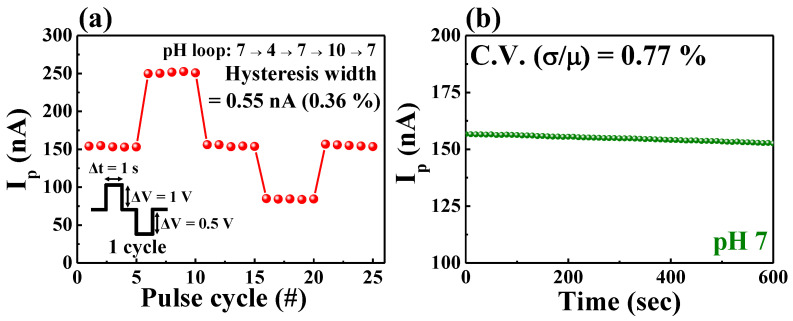
(**a**) Hysteretic and (**b**) drift responses of a time-dependent pH sensor by using a chitosan/Ta_2_O_5_ hybrid dielectric EDLT.

One of the fundamental requirements for a sensor is the reliability of maintaining a constant sensitivity for repetitive and prolonged sensing operations. In particular, for EG-ISFET-based pH sensors, changes in electrical properties and sensitivity may be affected by defects in the EG membrane or gate insulator of the transistor [36]. Therefore, we measured hysteresis and drift responses to evaluate the repeatability, reliability, and stability of pH sensors fabricated using a chitosan/Ta_2_O_5_ hybrid dielectric EDLT. The ion migration of the chitosan electrolyte enabled the unique pH sensitivity control, but the long-term memory effect induced by the migrated ions may lead to the deterioration of the stability and reliability. Hence, for initialization of this unwanted long-term memory effect, we used a pair of sensing pulses (1 V, 1 s) and erasing pulses (−0.5 V, 1 s) as one pulse cycle. Figure 7 shows the reliability and stability of the chitosan/Ta_2_O_5_ hybrid dielectric EDLT pH sensors evaluated subject to this condition. The hysteretic effect induced by repeatedly changing the pH buffers according to the hysteretic loop of pH 7 → 4 → 7 → 10 → 7 is shown in Figure 7a. The inset shows single cycles of gate pulses used for hysteresis and drift measurements. The difference in I_p_ values between the first and third pH 7 cycles (hysteretic width) is 0.55 nA, and the deviation is 0.38%, thus showing good repeatability and stability [37,38]. Figure 7b shows the drift response for I_p_ measured for 600 s in a pH 7 buffer solution, with no abrupt changes in I_p_ even after long-term operation. The coefficient of variation extracted from this result (based on the standard deviation (σ) and average (μ)) was 0.77%, with a good stability and reliability of less than 1% [39,40]. As a result, the pH sensor (which used an EDLT) with tunable sensitivity characteristics proposed in this study successfully demonstrated excellent repeatability, stability, and reliability values < 1%.

## 3. Materials and Methods

### 3.1. Chitosan Electrolyte Solution Synthesis

The chitosan electrolyte solution was prepared by dissolving chitosan powder and acetic acid. The 2 wt% chitosan powder (>75% deacetylated, Sigma-Aldrich, City, State (if applicable), Country) obtained from shrimp shell was dissolved in a 2 wt% acetic acid solution and diluted with deionized water. The mixture was blended at 50 °C at 800 revolutions per minute in a magnetic stirring system. The solution was then filtered through a polytetrafluoroethylene syringe filter with a 5 μm pore size to obtain a clear and homogeneous solution.

### 3.2. Fabrication of the Chitosan–Ta_2_O_5_ Hybrid EDLTs as Transducer

As a starting material, a p-type silicon wafer on which a 100 nm-thick SiO_2_ layer was thermally grown was cleaned by a standard Radio Corporation of America (RCA) process. A 50 nm-thick a-IGZO channel layer was deposited by radio frequency (RF) magnetron sputtering subject to the following conditions: RF power: 100 W; working pressure: 6 mTorr; and Ar gas flow: 30 sccm. The active area of the a-IGZO channel layer was defined by photolithography and a wet etching process by using a 30:1 buffer oxide etchant with channel length (L) and width (W) of 10 μm and 20 μm, respectively. A 150 nm-thick Ti source/drain electrode was deposited via an e-beam evaporator and patterned by a liftoff process. The prepared chitosan electrolyte solution was spin-coated on the devices, dried, and oven-baked at 130 °C for 10 min. Subsequently, an 80 nm-thick Ta_2_O_5_ barrier layer was deposited by RF magnetron sputtering to prevent chemical and mechanical damage, which enables a photolithography process for an organic chitosan electrolyte [41]. A 150 nm-thick Al layer was deposited by an e-beam evaporator and patterned through a lift-off process on the chitosan–Ta_2_O_5_ hybrid EDL gate dielectric as the top gate electrode. Finally, the source/drain contact holes were etched by a reactive ion etching system. The three-dimensional (3D) schematic and cross-sectional structure and the photograph of the fabricated chitosan/Ta_2_O_5_ hybrid dielectric EDLT are shown in Figure 1a and Figure 1b, respectively. In addition, to verify the unique sensing capability of the proposed pH sensor using EDLT, we also prepared normal FET devices with the same structure in which the gate dielectric (chitosan–Ta_2_O_5_ hybrid EDL) was replaced, with SiO_2_ with a thickness of 100 nm. A schematic of the cross-sectional structure of the normal FET and a photograph of the fabricated device are shown in Figure S1.

### 3.3. Fabrication of the Extended Gate as Sensing Membrane

The extended gate (EG) was fabricated on a transparent glass substrate (1.5 cm × 2.5 cm). A 300 nm-thick indium tin oxide (ITO) conductive layer was deposited by using RF magnetron sputtering. A 50 nm-thick SnO_2_ sensing membrane was deposited by RF magnetron sputtering subject to the following conditions: RF power: 50 W; working pressure: 3 mTorr; and Ar gas flow: 20 sccm. A polydimethylsiloxane (PDMS) reservoir with a diameter of 0.6 cm was attached to the SnO_2_ sensing membrane to inject and store the pH buffer solution. A photograph and the cross-sectional structure of the fabricated transparent EG are shown in Figure 1c.

### 3.4. Device Characterization

The current-voltage characteristics of the fabricated chitosan–Ta_2_O_5_ hybrid EDLT-based ISFET sensor were evaluated by using an Agilent 4156B precision semiconductor parameter analyzer. To evaluate the sensing behavior of the configured pH sensors, electrical pulses were applied to the EG by using an Agilent 8110A pulse generator. As a reference electrode, a commercially available Ag/AgCl electrode (Horiba 2080A-06T) was used for pH detection. All measurements were performed at room temperature, a relative humidity of ~25%, and the device was placed in a dark box during measurements to avoid electrical/optical noise.

## 4. Conclusions

In this study, a sensitivity tunable pH sensor composed of an EDLT with a chitosan/Ta_2_O_5_ hybrid gate dielectric was proposed. The mobile protons in the chitosan electrolyte of the EDLT migrated, owing to the gate voltage, and affected the I_D_. Because the electrical signal was transferred to the EG via a pH buffer solution, the number of migrated ions depended on the pH level and the gate pulse duration. This distinctive feature and dynamic sensing operation allowed the proposed pH sensor to easily adjust the sensitivity without additional gate electrodes or device-design changes. In EG-ISFETs composed of an SnO_2_ sensing membrane and EDLT, the maximum I_D_ (I_p_) response to gate pulses increased with decreasing pH level, and the growth rate of the I_D_ also improved as the pulse duration increased. Specifically, the sensitivity can be facilely adjusted, depending on the pulse duration, and can increase up to 174%, from 27.99 to 48.71 nA/pH. These characteristics were not observed in normal FETs (with SiO_2_ gate dielectrics) built for comparison, thus indicating that they are inherent in pH sensors with EDLT. We also evaluated the hysteresis and drift response of a pH sensor with EDLT, and demonstrated excellent repeatability, stability, and reliability values < 1%. As a result, the EG-ISFET-type pH sensor comprising the EDLT transducer is expected to be a useful biosensor platform with a simple tunable sensitivity and reliable sensing operation.

## Data Availability

Not applicable.

## Supplementary Materials

The supporting information can be downloaded at: https://www.mdpi.com/article/10.3390/ijms231810842/s1.

## References

[1] Manjakkal L., Szwagierczak D., Dahiya R. (2020). Metal oxides based electrochemical pH sensors: Current progress and future perspectives. Prog. Mater. Sci..

[2] Sinha S., Pal T. (2021). A comprehensive review of FET-based pH sensors: Materials, fabrication technologies, and modeling. Electrochem. Sci. Adv..

[3] Sinha S., Sahu N., Bhardwaj R., Ahuja H., Sharma R., Mukhiya R., Shekhar C. (2020). Modeling and simulation of temporal and temperature drift for the development of an accurate ISFET SPICE macromodel. J. Comput. Electron..

[4] Park J.K., Jang H.J., Park J.T., Cho W.J. (2014). SOI dual-gate ISFET with variable oxide capacitance and channel thickness. Solid-State Electron..

[5] Ma X., Peng R., Mao W., Lin Y., Yu H. (2022). Recent advances in ion-sensitive field-effect transistors for biosensing applications. Electrochem. Sci. Adv..

[6] Liu H.Y., Hsu W.C., Lee C.S., Chou B.Y., Chen W.F. (2015). Enhanced performances of AlGaN/GaN ion-sensitive field-effect transistors using H_2_O_2_-grown Al_2_O_3_ for sensing membrane and surface passivation applications. IEEE Sens. J..

[7] Lee T.N., Chen H.J., Hsieh K.C. (2016). Study on sensing properties of ion-sensitive field-effect-transistors fabricated with stack sensing membranes. IEEE Electron. Device Lett..

[8] Zeng Z., Wei W., Li B., Gao M., Chim W.K., Zhu C. (2022). Low Drift Reference-less ISFET Comprising Two Graphene Films with Different Engineered Sensitivities. ACS Appl. Electron. Mater..

[9] Könemund L., Neumann L., Hirschberg F., Biedendieck R., Jahn D., Johannes H.H., Kowalsky W. (2022). Functionalization of an extended-gate field-effect transistor (EGFET) for bacteria detection. Sci. Rep..

[10] Wei W., Liao W., Zeng Z., Zhu C. (2019). Extended gate reference-FET (REFET) using 2D h-BN sensing layer for pH sensing applications. IEEE Electron. Device Lett..

[11] Cho W.J., Lim C.M. (2018). Sensing properties of separative paper-based extended-gate ion-sensitive field-effect transistor for cost effective pH sensor applications. Solid-State Electron..

[12] Yuan H., Shimotani H., Tsukazaki A., Ohtomo A., Kawasaki M., Iwasa Y. (2009). High-density carrier accumulation in ZnO field-effect transistors gated by electric double layers of ionic liquids. Adv. Funct. Mater..

[13] He Y., Yang Y., Nie S., Liu R., Wan Q. (2018). Electric-double-layer transistors for synaptic devices and neuromorphic systems. J. Mater. Chem. C.

[14] Khademi M., Barz D.P. (2020). Structure of the electrical double layer revisited: Electrode capacitance in aqueous solutions. Langmuir.

[15] Saito S., Tamate R., Miwa K., Shimizu S., Horii T., Ueno K., Ono S., Watanabe M. (2020). High performance electric double layer transistors using solvate ionic liquids. Jpn. J. Appl. Phys..

[16] Huang H.Y., Ge C., Zhang Q.H., Liu C.X., Du J.Y., Li J.K., Wang C., Gu L., Yang G.Z., Jin K.J. (2019). Electrolyte-gated synaptic transistor with oxygen ions. Adv. Funct. Mater..

[17] Liu N., Chen R., Wan Q. (2019). Recent advances in electric-double-layer transistors for bio-chemical sensing applications. Sensors.

[18] Du H., Lin X., Xu Z., Chu D. (2015). Electric double-layer transistors: A review of recent progress. J. Mater. Sci..

[19] Cheng Q., Wang M., Tao M., Yin R., Li Y., Yang N., Xu W., Gao C., Hao Y., Yang Z. (2020). Planar dual gate GaN HEMT cascode amplifier as a voltage readout pH sensor with high and tunable sensitivities. IEEE Electron. Device Lett..

[20] Spanu A., Viola F., Lai S., Cosseddu P., Ricci P.C., Bonfiglio A. (2017). A reference-less pH sensor based on an organic field effect transistor with tunable sensitivity. Org. Electron..

[21] Pfattner R., Foudeh A.M., Chen S., Niu W., Matthews J.R., He M., Bao Z. (2019). Dual-Gate Organic Field-Effect Transistor for pH Sensors with Tunable Sensitivity. Adv. Electron. Mater..

[22] He Y., Wang X., Zhou J.Y., Wang T.T., Ren M.K., Chen G.Q., Pu T.F., Li X.B., Jia M., Bu Y.Y. (2021). Enhanced pH sensitivity of AlGaN/GaN ion-sensitive field-effect transistor by recess process and ammonium hydroxide treatment. IEEE. Trans. Electron. Devices.

[23] Cho Y.S., Kim S.K., Ahn C.B., Je J.Y. (2021). Preparation, characterization, and antioxidant properties of gallic acid-grafted-chitosans. Carbohydr. Polym..

[24] Indrani D.J., Lukitowati F., Yulizar Y. (2017). Preparation of chitosan/collagen blend membranes for wound dressing: A study on FTIR spectroscopy and mechanical properties. IOP Conf. Ser. Mater. Sci. Eng..

[25] Grande R., Carvalho A.J. (2011). Compatible ternary blends of chitosan/poly (vinyl alcohol)/poly (lactic acid) produced by oil-in-water emulsion processing. Biomacromolecules.

[26] Jiang S., He Y., Liu R., Zhang C., Shi Y., Wan Q. (2021). Synaptic metaplasticity emulation in a freestanding oxide-based neuromorphic transistor with dual in-plane gates. J. Phys. D Appl. Phys..

[27] Zhu L.Q., Wan C.J., Guo L.Q., Shi Y., Wan Q. (2014). Artificial synapse network on inorganic proton conductor for neuromorphic systems. Nat. Commun..

[28] Wen J., Zhu L.Q., Fu Y.M., Xiao H., Guo L.Q., Wan Q. (2017). Activity dependent synaptic plasticity mimicked on indium–tin–oxide electric-double-layer transistor. ACS Appl. Mater. Interf..

[29] Fu Y.M., Li H., Huang L., Wei T., Hidayati F., Song A. (2022). Sputtered Electrolyte-Gated Transistor with Modulated Metaplasticity Behaviors. Adv. Electron. Mater..

[30] Kim S.H., Cho W.J. (2021). Lithography processable Ta_2_O_5_ barrier-layered chitosan electric double layer synaptic transistors. Int. J. Mol. Sci..

[31] Yates D.E., Levine S., Healy T.W. (1974). Site-binding model of the electrical double layer at the oxide/water interface. J. Chem. Soc. Faraday Trans. 1 Phys. Chem. Condens. Phases.

[32] Pan T.M., Huang M.D., Lin C.W., Wu M.H. (2010). Development of high-κ HoTiO_3_ sensing membrane for pH detection and glucose biosensing. Sens. Actuators B Chem..

[33] Sinha S., Pal T., Sharma P., Kharbanda D., Khanna P.K., Tanwar A., Sharma R., Mukhiya R. (2021). Fabrication, Characterization, and Modeling of an Aluminum Oxide-Gate Ion-Sensitive Field-Effect Transistor-Based pH Sensor. J. Electron. Mater..

[34] Kaisti M., Zhang Q., Prabhu A., Lehmusvuori A., Rahman A., Levon K. (2015). An ion-sensitive floating gate FET model: Operating principles and electrofluidic gating. IEEE Trans. Electron. Devices.

[35] Jin B., Lee G.Y., Park C., Kim D., Choi W., Yoo J.W., Pyun J.C., Lee J.S. (2018). Electrical characteristics and pH response of a parylene-H sensing membrane in a Si-nanonet ion-sensitive field-effect transistor. Sensors.

[36] Bousse L., Bergveld P. (1984). The role of buried OH sites in the response mechanism of inorganic-gate pH-sensitive ISFETs. Sens. Actuators.

[37] Yang C.M., Zeng W.Y., Chen C.H., Chen Y.P., Chen T.C. (2018). Spatial resolution and 2D chemical image of light-addressable potentiometric sensor improved by inductively coupled-plasma reactive-ion etching. Sens. Actuators B Chem..

[38] Yang C., Chen C.-H., Akuli N., Yen T.H., Lai C.S. (2020). Chemical Illumination modification from an LED to a laser to improve the spatial resolution of IGZO thin film light-addressable potentiometric sensors in pH detections. Sens. Actuators B Chem..

[39] Slewa L.H., Abbas T.A., Ahmed N.M. (2019). Synthesis of quantum dot porous silicon as extended gate field effect transistor (EGFET) for a pH sensor application. Mater. Sci. Semicond. Process..

[40] Wu Y.C., Wu S.J., Lin C.H. (2015). High performance EGFET-based pH sensor utilizing low-cost industrial-grade touch panel film as the gate structure. IEEE Sens. J..

[41] Min S.Y., Cho W.J. (2020). CMOS-compatible synaptic transistor gated by chitosan electrolyte-Ta_2_O_5_ hybrid electric double layer. Sci. Rep..

