# Supplementary material for: Time-Dependent Sensitivity Tunable pH Sensors Based on the Organic-Inorganic Hybrid Electric-Double-Layer Transistor
Source: Int J Mol Sci. 2022 Sep 16;23(18):10842. doi: 10.3390/ijms231810842 (PMC9503050; doi:10.3390/ijms231810842)
Supplement: Supplementary file 1 [file ijms-23-10842-s001.zip › ijms-1881016-supplementary.pdf]

International Journal of *Molecular Sciences*

MDPI

Supplementary Information

Time-Dependent Sensitivity Tunable pH Sensors Based on the Organic-Inorganic Hybrid Electric-Double-Layer Transistor

Ki-Woong Park and Won-Ju Cho *

Department of Electronic Materials Engineering, Kwangwoon University, 447-1 Wolgye-dong, Nowon-gu, Seoul 139-701, Korea
* Correspondence: chowj@kw.ac.kr

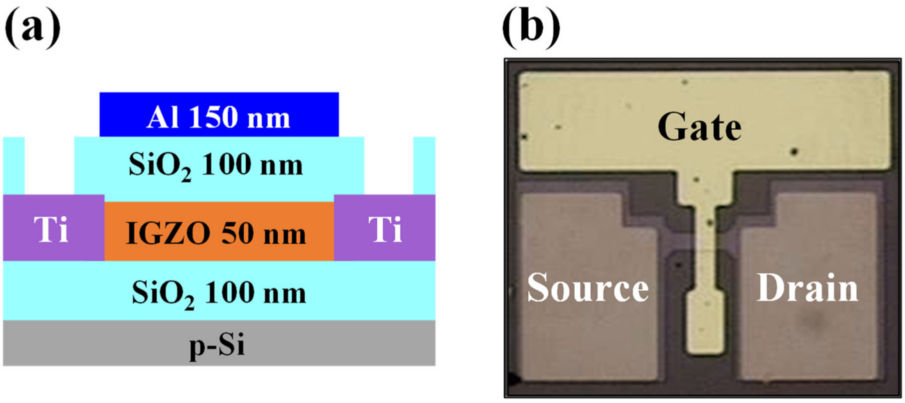

Figure S1. (**a**) Cross-section of structure. (**b**) Optical microscopy image of the fabricated normal field-effect transistor (FET) with SiO_2 gate dielectric.

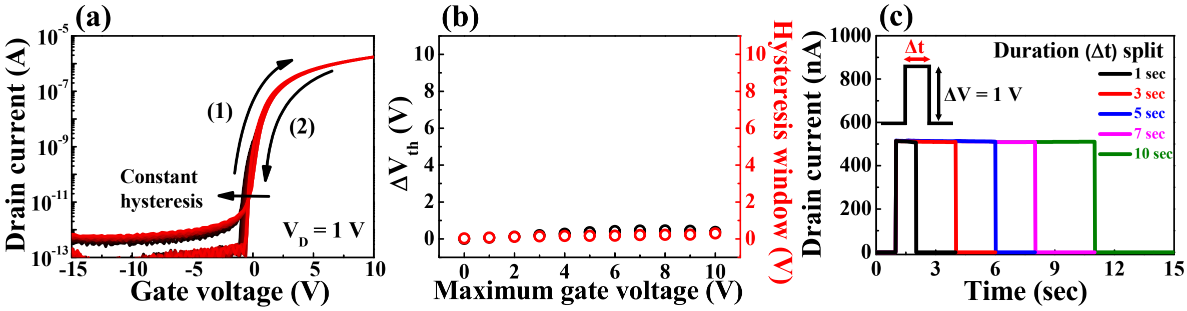

Figure S2. (**a**) Double sweep transfer curves of normal FETs with SiO_2 gate dielectric at increasing maximum V_G and (**b**) threshold voltage and hysteresis window extracted from transfer curves. (**c**) Dynamic I_D responses to gate pulses with various durations.

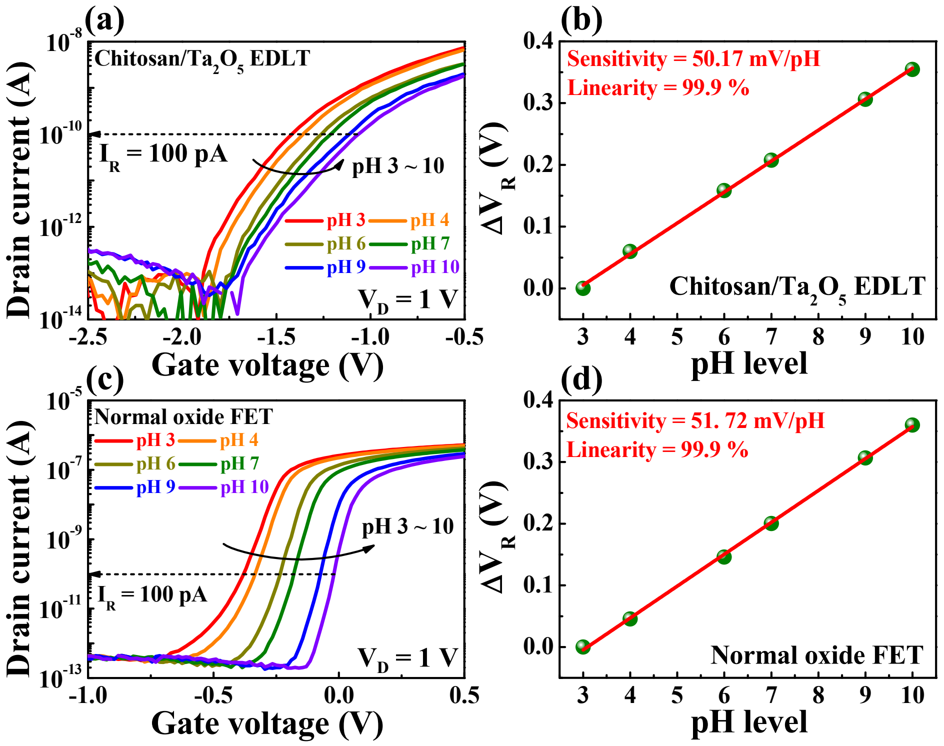

Figure S3. (**a**,**c**) Transfer curve shift and (**b**,**d**) reference voltage change in quasistatic sensing mode. (**a**,**b**) chitosan/Ta_2O_5 hybrid dielectric EDLTs. (**c**,**d**) normal FETs with SiO_2 gate dielectric.

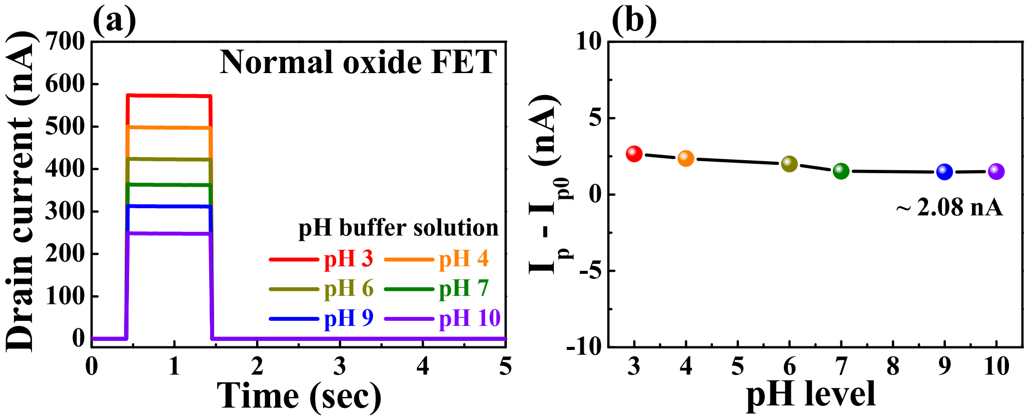

Figure S4. (**a**) Dynamic I_D responses in various pH buffer solutions, and (**b**) change of I_D (I_p - I_{p0}) subject to the application of a gate pulse (1s duration) in a normal FET with SiO_2 gate dielectric.

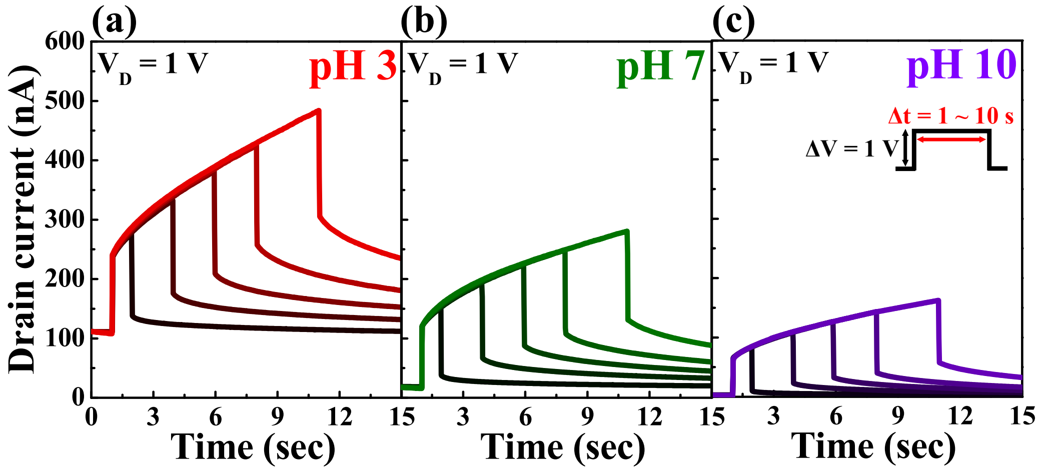

Figure S5. (**a**) Dynamic I_D responses st various sensing pulse duration in the chitosan/Ta_2O_5 hybrid dielectric EDLT at (**a**) pH 3, (**b**) pH 7, and (**c**) pH 10.

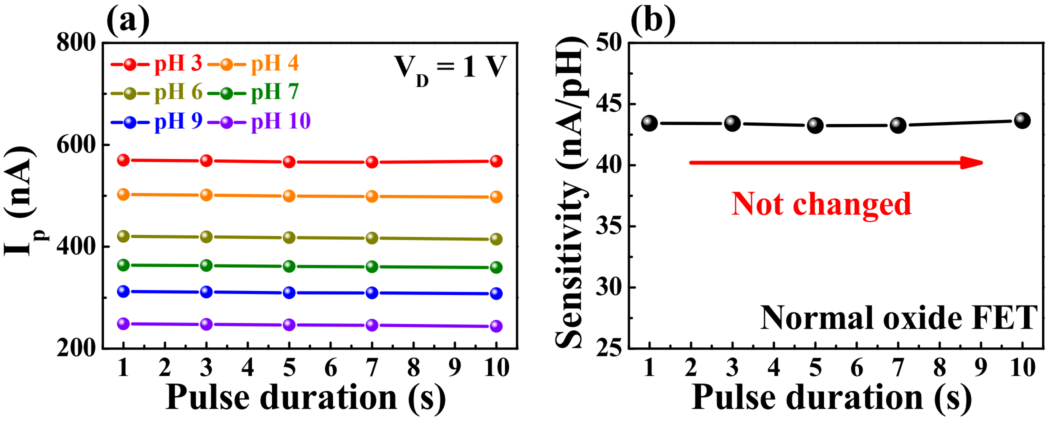

Figure S6. (**a**) Modulation of I_P for gate pulse and (**b**) pH sensitivity as a function of pulse duration in a normal FET with SiO_2 gate dielectric in various pH buffers.
